# Barriers and Facilitators to the Use of Novel Injectable Lipid-Lowering Therapies in Patients with Dyslipidemia or Cardiovascular Disease: A Scoping Review

**DOI:** 10.3390/medicina62050843

**Published:** 2026-04-28

**Authors:** Gabriele Caggianelli, Marco Iorfida, Renato Cavaliere, Alessandro Manzoli, Antonio D’Angelo, Francesco Scerbo, Flavio Marti, Stefano Mancin, Giovanni Cangelosi, Gennaro Rocco, Valentina Vanzi, Vineetha Karuveettil, Maurizio Zega, Clara Donnoli

**Affiliations:** 1 Department of Healthcare Professions, Azienda Ospedaliera San Giovanni Addolorata, 00184 Rome, Italyiorfida.m@gmail.com (M.I.);; 2 JBI Italy Evidence-Based Practice and Health Research Center, 00146 Rome, Italy; 3 School of Nursing and Midwifery, Sapienza University of Rome, Azienda Ospedaliera San Camillo-Forlanini Hospital, 00152 Rome, Italy; 4 School of Nursing, Sapienza University of Rome, 00185 Rome, Italy; 5 Center of Excellence for Nursing Culture and Research, Order of Nursing Professions of Rome, 00165 Rome, Italy; scerbofrancesco@gmail.com (F.S.);; 6 IRCCS Humanitas Research Hospital, 20089 Rozzano, Italy; 7 School of Pharmacy, Experimental Medicine and “Stefania Scuri” Public Health Department, 62032 Camerino, Italy; 8 International Center for Nursing Research Montianum Our Lady of Good Counsel Catholic, University of Tirana, 1000 Tirana, Albania; 9 Department of Public Health Dentistry, Amrita School of Dentistry, Amrita Vishwa Vidyapeetham, Kochi 682041, Kerala, India; 10 JBI Amrita Centre for Evidence Synthesis and Implementation, Kochi 682041, Kerala, India; 11 Nursing Professions Board of Rome, 00161 Rome, Italy

**Keywords:** dyslipidemia, cardiovascular disease, lipid-lowering therapy, proprotein convertase subtilisin/kexin type 9 (PCSK9) inhibitors, medication adherence, scoping review

## Abstract

*Background/Aim*: Cardiovascular disease (CVD) represents a relevant global public health challenge with dyslipidemia as a major modifiable cardiovascular risk factor (CVRF). Recent advances have introduced injectable lipid-lowering therapies (LLT). Their clinical effectiveness in real-world practice seems to depend not only on pharmacological efficacy but also on patients’ acceptance, adherence, and persistence, influenced directly by perceived barriers and facilitators. The main objective of this scoping review is to map the barriers and facilitators related to the use of novel injectable LLTs among adult patients with dyslipidemia or CVD. *Methods*: This review was conducted in accordance with JBI methodology and reported according to Preferred Reporting Items for Systematic reviews and Meta-Analyses Extension for scoping reviews (PRISMA-ScR); pre-registration on Open Science Framework (OSF) was performed. A search was conducted in MEDLINE from PubMed, Cochrane Library, Cumulative Index to Nursing and Allied Health Literature (CINAHL) from EBSCOhost, and Google Scholar up to June 2025. Eligible studies included qualitative, quantitative, mixed-methods, and review papers involving adult patients with dyslipidemia who reported experiences, perceptions or challenges related to the use of injectable LLT in any healthcare or community setting worldwide. Two reviewers independently screened studies, selected and extracted data. *Results*: Out of 665 records identified, 7 studies met the inclusion criteria. Patients’ adherence to injectable LLTs is shaped by psychological fears, prior negative experiences, and perceived efficacy. Satisfaction increases when patients feel supported and informed. Convenience, self-administration, and motivational meaning facilitate persistence. Organizational support and economic accessibility further influence uptake, highlighting that adherence depends on both patient experience and structural factors. *Conclusions*: Patient acceptance and persistence with injectable LLT depends on a complex interplay of emotional, clinical, organizational and economic factors, beyond pharmacological efficacy alone. Fear of injections, previous statin-related experiences, administrative complexity, and high costs remain major barriers, while shared decision-making, trust in healthcare providers, perceived efficacy, regimen convenience, and supportive structures act as strong facilitators. Addressing these challenges requires multidimensional and multidisciplinary strategies for policy makers and clinical managers.

## 1. Introduction

Cardiovascular disease (CVD) is a leading global public health concern, [1,2,3] and dyslipidemia represents a major modifiable cardiovascular risk factor (CVRF). According to the 2025 Focused Update of 2019 European Society of Cardiology (ESC) and European Atherosclerosis Society (EAS) guidelines, patients presenting with clinically or imaging-documented atherosclerotic CVD, diabetes, severe renal impairment, or at least one poorly controlled major risk factor are classified as being at high or very high CVRF [4]. Lipid-modifying agents (LMAs) are strongly recommended for reducing atherogenic lipid levels and for the prevention of coronary artery disease events in several populations with or without established CVD [5,6,7]. Analysis of pharmaceutical sales data covering 83 countries estimated that approximately 173 million individuals were treated with LMAs in 2018, reflecting a compound annual growth rate of 4.13% from 2008 to 2018. Statins have long represented the cornerstone of lipid-lowering therapy worldwide [8]. However, despite the substantial improvements in CVD outcomes associated with statin therapy, up to 40% of patients receiving intensive statin treatment continue to experience major CVD events even after achieving Low-Density Lipoprotein Cholesterol (LDL-C) targets, a phenomenon commonly referred to as “residual risk” [9]. In response to the persistence of residual risk, innovative therapeutic strategies have been developed, reflecting the evolving landscape of lipid management in CV health [10]. Recent advances in pharmacology, combined with a deeper understanding of atherogenesis, have led to the identification of novel therapeutic targets and the development of potent new agents, including monoclonal antibodies proprotein convertase subtilisin/kexin type 9 (PCSK9) inhibitors (evolocumab, alirocumab), small interfering RNA interferer (inclisiran) and bempedoic acid. These therapies have demonstrated effectiveness in intensifying lipid-lowering treatment, achieving LDL-C goals, and mitigating statin-associated adverse effects, such as myopathy [11,12,13]. However, the translation of these pharmacological advances into meaningful cardiovascular benefit depends not only on their efficacy and safety, but also on their successful integration into patients’ daily care routines [14]. In this context, the concepts of barriers and facilitators are central to understanding treatment uptake and adherence. Barriers are defined as individual, interpersonal, or systemic factors that hinder patients’ ability or willingness to initiate and maintain therapy, whereas facilitators are factors that support, enable, or motivate successful engagement with treatment [15,16,17]. Barriers and facilitators may arise from patient-level determinants as well as organizational and structural factors and their interaction influences not only adherence but also the achievement of intended clinical outcomes, underscoring the importance of incorporating patient perspectives into the implementation of new therapies [18,19]. In this regard, the introduction of new injectable LLTs, including PCSK9 inhibitors and inclisiran, presents unique considerations due to differences in administration, dosing frequency, and care delivery models compared with traditional oral therapies. PCSK9i monoclonal antibodies are typically administered via subcutaneous injection every 2 to 4 weeks, requiring either clinic visits or patient self-injection training, whereas inclisiran is administered subcutaneously at baseline, 3 months, and then every 6 months thereafter [20,21]. While the less frequent dosing schedule of inclisiran may facilitate adherence, it also requires consistent engagement with healthcare services for administration, from clinical and nursing perspectives [22,23]. Globally, the adoption of injectable LLT is increasing, although substantial heterogeneity remains. Higher uptake is observed in European and North American populations, particularly among patients with established Atherosclerotic Cardiovascular Disease (ASCVD) or familial hypercholesterolemia, whereas access is limited in low- and middle-income countries due to availability, cost, and reimbursement barriers [24]. Economic considerations, including the higher acquisition cost of injectable therapies relative to generic statins, alongside insurance coverage limitations, represent potential obstacles to initiation and persistence, even in high-income settings [25]. Such a multidimensional and multidisciplinary strategy has been recognized as essential for reducing the burden of CVD, by integrating expertise from physicians, nurses, and other allied professionals to address complex risk profiles and optimize patient outcomes [26]. Despite the increasing use of injectable LLTs, less is known about patient experiences, perceived barriers, and facilitators influencing their uptake and persistence in real-world settings.

### Aim and Research Question

The present scoping review aims to map the barriers and facilitators related to the use of novel injectable LLTs among adult patients with dyslipidemia or CVD. The review would answer following question:

What barriers and facilitators related to the use of novel injectable LLTs (PCSK9 inhibitors and inclisiran) are reported in the literature among adult patients with dyslipidemia or CVDs?

## 2. Methods

The scoping review was conducted in accordance with the JBI methodology for scoping reviews [27] and reported according to the Preferred Reporting Items for Systematic reviews and Meta-Analyses Extension for scoping reviews (PRISMA-ScR) reporting guideline (Checklist in Supplementary File S1) [28]; pre-registration in Open Science Framework (OSF) was performed (https://osf.io/m8p9j accessed 23 February, 2026).

### 2.1. Inclusion Criteria

Inclusion and exclusion criteria were structured according to the Participant, Concept, Context (PCC) framework [27].

#### 2.1.1. Participants

The population of interest includes adult patients (≥18 years) with dyslipidemia and/or cardiovascular disease or cardiovascular risk factors who are eligible for, or have experience with, LLT. Papers regarding pediatric populations or non-human studies were excluded. No restrictions were applied regarding gender, ethnicity, or geographical location, in order to capture evidence across diverse patient groups.

#### 2.1.2. Concept

The concept focuses on barriers (individual, interpersonal, or systemic factors that may limit the initiation, adherence, or continuation of therapy) and facilitators (factors that support or promote treatment engagement) related to the use of injectable LLTs, including PCSK9 inhibitors such as evolocumab and alirocumab, as well as inclisiran. Studies examining oral lipid-lowering agents exclusively (e.g., statins, ezetimibe, or bempedoic acid) were excluded unless they also addressed injectable therapies. Articles reporting exclusively the perspectives of healthcare professionals or policymakers, or focusing solely on pharmacological efficacy, safety, or cost-effectiveness, were also excluded.

#### 2.1.3. Context

The context encompasses any healthcare or community setting, including primary care, specialist outpatient clinics, hospitals, nursing community care and broader community-based care, without restrictions on healthcare system or country organization.

### 2.2. Types of Sources

This scoping review considered both experimental and quasi-experimental study designs including randomized controlled trials, non-randomized controlled trials, before and after studies and interrupted time-series studies. In addition, analytical observational studies including prospective and retrospective cohort studies, case-control studies and analytical cross-sectional studies were considered for inclusion. This review also considered descriptive observational study designs including case series, individual case reports and descriptive cross-sectional studies for inclusion. Qualitative studies were also considered that focus on qualitative data including, but not limited to, designs such as phenomenology, grounded theory, ethnography, qualitative description, action research and feminist research. In addition, systematic reviews that met the inclusion criteria were also considered, according mainly to the research question. Conference acts and opinion papers were also considered for inclusion in this scoping review.

### 2.3. Search Strategy

The search strategy aimed to locate both published and unpublished studies. A three-step search strategy was adopted in this review. First, an initial limited search of MEDLINE via PubMed and Cumulative Index to Nursing and Allied Health Literature (CINAHL) from EBSCOhost was undertaken to identify articles on the topic. The text words contained in the titles and abstracts of relevant articles, and the index terms used to describe the articles were used to develop a full search strategy for MEDLINE from PubMed, Cochrane Database of Systematic Reviews (Cochrane Library), CINAHL from EBSCOhost and Google Scholar (Search Strategy in Supplementary File S2). The search strategy, including all identified keywords and index terms, was adapted for each included database and information source. The reference list of all included sources of evidence was screened for additional studies. Studies published in English and Italian were considered for inclusion. In addition, the authors had reserved the right to include studies published in other languages provided that the title and/or abstract was available in English and suggested the study meeting the inclusion criteria, however it did not occur.

### 2.4. Source of Evidence Selection

Following the search, all identified citations were collated and uploaded into the web-based tool Rayyan [29] and duplicates removed. Following a pilot test, titles and abstracts were independently screened by two independent reviewers for assessment against the inclusion criteria for the review. The full text of selected citations was assessed in detail against the inclusion criteria independently by the reviewers. Reasons for exclusion of sources of evidence at full text that did not meet the inclusion criteria were recorded and reported. Any disagreements that arose between the reviewers at each stage of the selection process were resolved through discussion. For citation management the software Zotero (vers. 7.0.11 or later) was used.

### 2.5. Data Extraction and Analysis

Data extraction and analysis were conducted by one reviewer and accuracy was checked by a second reviewer. Data were extracted from sources of evidence included in the scoping review using a reviewer-developed data extraction form in Microsoft Word (Redmond, WA, USA), based on the JBI template described in the JBI Manual for Evidence Synthesis [27]. The form was piloted on a small sample of studies, refined as needed, and subsequently applied to all included studies. The data extraction tool was refined throughout the process to ensure all extracted data were accounted for. The extracted data included: authors, year, nation, study design, outcome, pharmacological therapy and adherence results. Following data extraction, the information gathered from the included studies was examined through a thematic analytical process inspired by the approach described by Braun and Clarke (2006) [30]. This process involved organizing and interpreting the extracted data through successive steps of grouping related elements, mapping recurring patterns, and categorizing them into broader themes. As an initial step, studies and their extracted items were organized according to the general characteristics defined in the data extraction form. This preliminary organization provided a structured basis for identifying relationships among the data and for exploring emerging thematic patterns across the included publications. To provide a clearer synthesis of the findings, tables were created to summarize the extracted data. These findings were synthesized into four main domains: psychological and emotional (fear of injections, trust, shared decision-making, overall satisfaction), clinical experience and treatment perception (side effects, impact on daily life, convenience, stability, and efficacy), motivational dimension (personal meaning, convenience), and organizational/access-related aspects and economic dimension (bureaucracy, coverage, community support, cost, sustainability). Any disagreements that arose between the reviewers were resolved through discussion. No authors were contacted to request additional data.

## 3. Results

### 3.1. Selection Process

**Figure 1:**
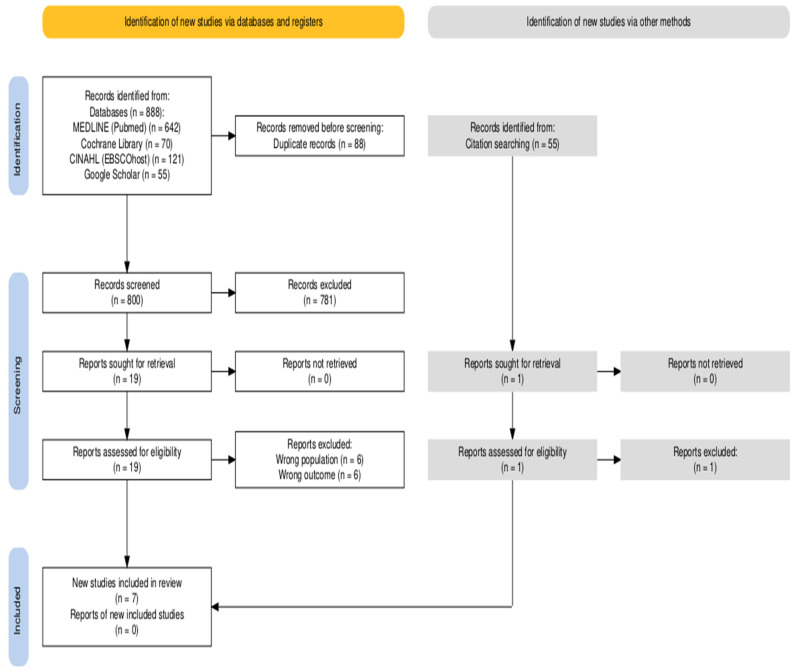
PRISMA Flow chart.

From search strategy, 888 records screened and 55 found through hand search. After the exclusion of 88 duplicates, 800 titles were screened. A total of 781 titles and abstracts were excluded according to the predefined criteria, leaving 19 full-text articles from databases and one from hand search assessed for eligibility. Of these, 13 did not meet the inclusion criteria. The final selection included 7 studies [31,32,33,34,35,36,37] published between 2017 and 2025 (Figure 1).

### 3.2. General Characteristics of Included Studies

The most represented country of origin is the United States (US) [31,32]; there are also contributions from the United Kingdom (UK) [35], Netherlands [37] and Canada [36]; one study was conducted between the UK and Italy [34]. The included studies were methodologically heterogeneous. Two were reviews focusing on patient adherence, perceptions, and access barriers [31,32]; two were descriptive qualitative studies investigating patients’ experiences, perceptions, facilitators, and barriers to injectable LLTs [34,35]; one was a retrospective cohort study evaluating real-world effectiveness and treatment persistence [36]; one was a patient survey exploring perceived barriers to PCSK9i use [33]; and one adopted a mixed-methods design examining patient preferences and the role of shared decision-making [37] (Summary in Table 1).

**Table 1:** Characteristics of included studies.

Authors	Year	Nation	Study Design	Outcome	Pharmacological Therapy	Adherence Results|Barriers|Facilitators
Mulder et al. [37]	2025	The Netherlands	Mixed methods	Analyzing patient experiences/preferences and role of shared decisions in the use of PCSK9i	PCSK9i (mAb and siRNA included inclisiran)	High satisfaction (96%); high perceived efficacy (83%)|Needle phobia (~5%)|Shared decision-making; perceived benefit
Baig et al. [35]	2024	UK	Descriptive qualitative study	Exploring clinicians’ perception of patient behaviors/experiences regarding new injectable LLT	PCSK9 (included inclisiran)	Not reported|Injection rejection; needle phobia; distrust of new drugs|Not reported
Siemens et al. [36]	2024	Canada	Retrospective cohort study	Evaluating real efficacy and patient-reported reasons for discontinuation	Alirocumab, Evolocumab (PCSK9 mAb)	Majority maintained therapy|Cost; adverse events; temporary interruptions; non-adherence|Perceived clinical effectiveness
Lee et al. [34]	2023	UK/Italy	Descriptive qualitative study	Exploring patient perceptions and experiences about facilitators and barriers to the use of injectable therapies	PCSK9i (mAb and siRNA)	Not reported|Fear of injections; practical/logistic burden|Clinical support; simple regimen; perceived benefits
Wong et al. [33]	2021	USA	Survey	Exploring perceived barriers to the use of PCSK9i in patients	PCSK9 (alirocumab, evolocumab)	Not reported|Insurance/cost barriers; access issues; injection hesitancy|Physician recommendation
Kosmas et al. [31]	2018	USA	Review	Summarize patient adherence, compliance and outlook on evolocumab	Evolocumab (PCSK9 mAb)	Very high adherence (~95% successful self-injection)|Minimal|Ease of use; convenience; better acceptance than statins
Baum et al. [32]	2017	USA	Review	Describe access barriers and patient perceptions on PCSK9i	PCSK9i (alirocumab, evolocumab)	Not reported|High initial denial rates (80–90%); access restrictions|Coverage approval when granted

Legend. PCSK9: Proprotein Convertase Subtilisin/Kexin type 9; mAb: monoclonal antibody; siRNA: small interfering ribonucleic acid; LLT: Lipid-Lowering Therapy.

### 3.3. Clinical and Care-Related Findings

The therapies investigated were predominantly monoclonal antibody PCSK9 inhibitors (alirocumab and evolocumab), while some studies also referred to inclisiran as part of the broader class of novel injectable LLT [35,37]. The retrospective cohort study reported high real-world effectiveness, with most patients achieving clinically relevant LDL-C reductions over 12 months of therapy, and overall adherence described as generally good. Nevertheless, cases of non-adherence and interruptions have also been reported, mostly due to unsustainable costs or adverse events, such as myalgia, fatigue and local injection-site reactions [36]. Qualitative studies highlighted patients’ subjective experiences with injectable lipid-lowering therapies. Commonly reported initial barriers included fear of injections or needles, anxiety related to previous statin experience, and logistical concerns (storage requirements and travel concerns) [34]. At the same time, patients reported positive perceptions linked to the strong clinical effectiveness of PCSK9 inhibitors, particularly visible reduction in LDL-C values, which were perceived as tangible indicators of benefit [31,34,37]. The reduced frequency of administration was described as convenient compared with daily oral regimens. Trust in clinicians and feeling supported during treatment initiation were facilitating elements. From the perspective of healthcare professionals and system-level analyses, several studies described significant access-related barriers. Reviews and surveys highlighted difficulties in accessing the drugs, characterized by very high initial insurance denials (up to 80–90%), complex bureaucratic procedures, and concerns about long-term economic sustainability [31,32]. Barriers and facilitators are summarized in Table 2 and Table 3.

### 3.4. Thematic Analysis

From the thematic analysis, 22 categories emerged which were organized into 4 main themes (Summary Table 4).

#### 3.4.1. Psychological and Emotional Dimension

Fear and anxiety surrounding injections were among the most consistently reported psychological barriers. Patients expressed apprehension about self-administration, possible pain, and concerns about handling the devices correctly [34,35,37]. Prior negative experiences with LLT, especially statins, shaped these concerns, with some patients carrying forward expectations of adverse effects or failure [33,37]. Despite these anxieties, overall satisfaction with injectable therapies was generally high once treatment was initiated, particularly when patients experienced clinical benefit and were supported by their healthcare team [37]. Trust in physicians and nurses, as well as the presence of shared decision-making, emerged as crucial facilitators of acceptance. Studies emphasized that when patients felt listened to, informed, and supported, fears were reduced and adherence strengthened [34,35,37]. The emotional burden associated with injections or past treatment failures does not disappear entirely but appears to become more manageable when embedded in a supportive clinical relationship [34,36].

#### 3.4.2. Clinical Experience and Treatment Perception

Perceptions of safety and tolerability strongly influenced uptake and continuation. Reported side effects included myalgia, fatigue, and gastrointestinal symptoms, which echoed prior negative experiences with statins and occasionally limited enthusiasm for injectables [34,35,37]. However, many patients described minimal daily life disruption once established on treatment [34,37]. Positive experiences with self-injection were frequently noted. In both qualitative and clinical studies, patients reported that autoinjector devices were easy to use, convenient, and quickly integrated into routines [31,34]. A reduced frequency of administration, particularly with longer-interval dosing, was highlighted as a facilitator of adherence and treatment satisfaction [31,34,35,37]. Stability of the therapeutic regimen over time reinforced patient confidence and adherence [31,37]. Above all, perceived efficacy was the strongest motivator: patients and clinicians alike emphasized LDL-C reduction and Cardiovascular (CV) risk lowering as the most important reasons to accept and persist with therapy [31,34,37]. Importantly, this domain highlights how adherence is closely tied to patients’ perception of tangible benefit. Visible improvements in lipid values acted as reinforcing feedback, supporting persistence even in the presence of minor inconveniences or mild adverse effects [31,36,37].

#### 3.4.3. Motivational Dimension

Injectables carried significant motivational meaning. For many, they symbolized an alternative to ineffective or intolerable statins, offering renewed hope in therapy [31,32,34,35]. Patients also expressed a sense of being cared for and supported when offered innovative treatments, which reinforced adherence. Convenience—fewer pills and simplified regimens—was further identified as a motivational facilitator. Within this dimension, adherence appears linked to the meaning patients attribute to therapy. When injectables were perceived as innovative, effective, and tailored solutions after previous treatment limitations, patients described greater readiness to continue therapy despite its injectable nature [31,34,37].

#### 3.4.4. Organizational/Access-Related Aspects and Economic Dimension

Organizational support structures, including coordinated follow-up and involvement of patient communities, were shown to facilitate acceptance and adherence [32,34]. However, multiple studies highlighted substantial barriers related to reimbursement and insurance. Access restrictions and limited coverage were major challenges, often determining which patients ultimately received therapy [32,36]. Complex bureaucracy, prior authorization requirements, and delays in approval were repeatedly cited as obstacles, creating frustration for both patients and providers [31,32,33]. High costs, questions of long-term sustainability, and payer restrictions were consistently identified as barriers across settings [31,32,33,36]. Patients and providers noted that financial strain could delay initiation or force discontinuation, undermining clinical benefit. This domain illustrates how adherence is not solely an individual patient behavior but is also structurally conditioned. Even when patients were motivated and clinically appropriate for therapy, access restrictions and economic pressures could interrupt treatment continuity [31,32,33,36].

## 4. Discussion

This scoping review highlights that acceptance, adherence, and persistence with injectable LLT among patients with CVRF are influenced by a combination of psychological, clinical, motivational, and organizational factors. These elements interact throughout the care continuum, suggesting that therapeutic efficacy, although central, is insufficient to ensure the sustained use of these therapies in real-world practice. This perspective aligns with recent literature on adherence in chronic CV conditions, which conceptualizes treatment behavior as also dependent on the quality of care and the organization of health services [38,39]. From a psychological and emotional standpoint, fear of injections and anxiety related to self-administration frequently emerge as barriers, often amplified by prior negative experiences [40]. However, these responses are not fixed and can be mitigated when patients are engaged in care pathways characterized by continuity, clear information, and relational support [41]. Recent qualitative studies indicate that therapeutic education, counseling, and opportunities to engage with healthcare professionals help reduce uncertainty and strengthen confidence in treatment [42]. From a public health perspective, such interventions can be viewed as tools to reduce inequalities in access to and utilization of innovative therapies, transforming individual emotional barriers into systemically addressable care needs [43,44]. Clinical experience and treatment perception constitute a second key dimension. Evidence shows that perceived tolerability and, particularly, the visibility of clinical benefits play a crucial role in supporting treatment persistence; LDL-C reduction is frequently interpreted by patients as tangible evidence of treatment effectiveness, serving as positive reinforcement [31,32,33,34,35,36,37]. In this context, clinical monitoring and feedback extend beyond purely clinical evaluation, functioning as educational and motivational tools. The simplicity of administration devices and the low frequency of injections also reduce the care burden and facilitate the integration of therapy into daily routines, which is particularly relevant for the long-term management of chronic conditions [45,46]. Motivational aspects are closely linked to the meaning patients attribute to injectable therapies. For many, these treatments represent an effective alternative following prior therapeutic failures or intolerances, enhancing the sense of personalized care [47]. This symbolic dimension helps rebuild trust in the care pathway and healthcare system, sustaining patient engagement over time. Recent literature emphasizes that motivation should not be reduced to an individual trait but regarded as a relational construct developed through interactions with healthcare professionals and the organization of services [48]. Finally, organizational and economic factors emerge as structural determinants of adherence [49]. Bureaucratic complexity, prior authorization requirements, and reimbursement limitations constitute significant barriers capable of interrupting therapy even in motivated and clinically eligible patients [50]. These challenges underscore that adherence is not solely an individual responsibility but also an indicator of the healthcare system’s capacity to provide accessible, coordinated, and sustainable care [51]. In this context, healthcare professionals play a central role in mediating between clinical needs, administrative constraints, and available resources, contributing to the continuity of care [52]. Collectively, these findings indicate the need for multidimensional implementation strategies, in which therapeutic education, the continuity of care, and organizational simplification are integrated into cardiovascular prevention models [53]. A public health-oriented and patient-centered approach can promote more equitable and effective use of injectable LLT, ultimately improving health outcomes among populations at CVRF [54]. A useful comparison can be made with other injectable therapies, particularly glucagon-like peptide-1 receptor agonists (GLP-1 RAs) used in diabetes [55]. Unlike PCSK9 inhibitors, whose benefit is mainly reflected by biochemical feedback (LDL-C reduction), GLP-1 RAs provide more immediate clinical feedback, such as weight loss, which may enhance adherence, although with a less favorable tolerability profile, particularly due to gastrointestinal adverse effects [56]. In addition, prior experience with injectable therapies appears to reduce barriers to treatment initiation, as patients already receiving GLP-1 RAs may more readily accept the introduction of an additional injectable therapy such as PCSK9 inhibitors [57].

### 4.1. Implications for Clinical and Nursing Practice

To translate the findings of our study into clinical practice, future research should focus on specific strategies and interventions that support patients in the acceptance and management of injectable LLT, with a central role for nurses—an involvement already well established in the care of chronic conditions more broadly [58,59]. A first concrete area concerns the longitudinal assessment of therapeutic adherence, where nurses could implement structured follow-up protocols to detect changes over time in emotional barriers, treatment perceptions, and economic concerns, using validated tools to assess adherence and patient experiences [60]. These protocols may include regular contacts through modern, technologically advanced platforms, routine assistive follow-up, and shared monitoring sheets coordinated with the multidisciplinary team [61]. A second relevant area involves the implementation of educational and emotional support interventions specifically designed for patients initiating injectable therapies. Nurses could conduct personalized education sessions addressing injection-related fears, providing feedback on clinical effects, and offering strategies to integrate treatment into daily life, with the aim of enhancing patient competence and confidence [62].

### 4.2. Strengths and Limitations

The JBI methodology for scoping reviews followed by this review, ensures a transparent and systematic process for study selection, data extraction, and thematic synthesis. However, important limitations should be acknowledged. The number of eligible studies was small, reflecting the novelty of the topic and limiting the generalizability of findings. Study designs were heterogeneous, with qualitative data relying on relatively small patient samples, while reviews and surveys often reflected clinician or expert perspectives rather than direct patient voices. Most included studies originated from North America and Europe, meaning that barriers and facilitators relevant to low/middle-income countries remain largely unexplored. In addition, the rapid evolution of LLT (e.g., the introduction of inclisiran) means that findings from earlier studies on PCSK9 monoclonal antibodies may not fully capture patient experiences with newer drugs. Finally, no study provided long-term, prospective data on adherence trajectories, discontinuation reasons, or how perceptions evolve over time. Larger and more representative qualitative and mixed-methods studies are needed to explore patient perspectives across different healthcare systems, including underrepresented populations in low/middle-income countries. Future international research should evaluate longitudinal adherence and persistence with injectable LLT, examining how psychological barriers, clinical experiences, and economic concerns evolve over time from a multidisciplinary perspective.

## 5. Conclusions

This scoping review mapped the available evidence on challenges perceived by patients using new injectable LLT. Fear of injections, previous negative experiences with statins, side effects, complex bureaucratic procedures, and high costs were consistently identified as barriers. In contrast, shared decision-making, trust in healthcare professionals, perceived efficacy, ease of use, reduced frequency of administration, and supportive organizational structures facilitated therapy acceptance and adherence. These findings highlight that the successful integration of injectable LLT into clinical practice depends on addressing not only pharmacological efficacy but also patients’ lived experiences and the structural contexts in which these treatments are delivered. Interventions aimed at improving patient education, strengthening collaborative decision-making, reducing administrative and financial obstacles, and fostering community and organizational support are essential to maximize adherence and persistence. Ultimately, a patient-centered and system-sensitive approach will be critical to ensure that the proven CV benefits of these innovative therapies are fully realized in everyday practice.

## Figures and Tables

**Table 2:** Identified facilitators.

Authors (Year)	Positive Auto- Injection	Reduced Frequency/Comfort	Stability	Perceived Efficacy	Overall Satisfaction	Shared Decision-Making/Physician-Nurse Trust	Organizational/Community Support	Access/ Coverage	Personal Meaning
Mulder et al. (2025) [37]		X	X	X	X	X			
Baig et al. (2024) [35]		X				X			X
Siemens et al. (2024) [36]				X				X	
Lee et al. (2023) [34]	X	X		X		X	X		X
Kosmas et al. (2018) [31]	X	X	X	X					X
Baum et al. (2017) [32]				X			X	X	X

**Table 3:** Identified barriers.

Authors (Year)	Fear/Anxiety from Injection	Inadequate Information/Communication	Perceived Side Effects	Daily Life Impact	Previous Negative Experiences	Cost/ Sustainability	Bureaucracy and Access
Mulder et al. (2025) [37]	X		X	X	X		
Baig et al. (2024) [35]	X	X					
Siemens et al. (2024) [36]			X			X	
Lee et al. (2023) [34]	X	X	X	X	X		
Wong et al. (2021) [33]						X	X
Kosmas et al. (2018) [31]						X	X
Baum et al. (2017) [32]						X	X

**Table 4:** Emerged themes.

Main Theme	Included Categories	Emerged Contents (Barriers and Facilitators)
1. Psychological and emotional dimension	- Fear/anxiety about injection - Anxiety from previous experiences (statins) - Anxiety about familial/genetic risk - Trust in physician/nurse - Shared decision-making - Overall satisfaction	Barriers: needle phobia, initial anxiety, fear that statin symptoms will return, familial anxiety. Facilitators: trust in clinicians, shared decision, global satisfaction with therapy.
2. Clinical experience and treatment perception	- Perceived effectiveness - Perceived side effects (myalgia, fatigue, diarrhea, etc.) - Impact on daily life - “Fail-first therapy” stigma - Positive self-injection/ease of use - Stability of therapeutic regimen - Frequency/convenience of administration	Barriers: side effects (myalgia, fatigue, diarrhea, flu-like symptoms), perception of having to “fail first” with other therapies, impact on work/activity, travel/refrigerator logistics. Facilitators: strong perception of LDL-C efficacy, simple perceived auto-injection (95% success), reduced frequency (every 2 weeks, monthly, every 6 months), stable regimen, convenience compared to daily pills.
3. Motivational dimension	- Personal/motivational meaning (alternative to statins, hope in innovation, feeling cared for) - Positive reception among statin-intolerant patients - Perceived convenience (fewer pills)	Facilitators: remote monitoring perceived as reassuring, positive alternative for statins-intolerants, hope related to innovation, convenience of having fewer pills, sense of “doing the best” for one’s own health.
4. Organizational/access-related aspects and economic dimension	- Complex bureaucratic/insurance process - Initial denials/long waiting times - Access/appeal won/public coverage - High costs/sustainability - Organizational support (pandemic, follow-up) - Community support (associations, FH registries)	Barriers: insurance denials (80–90%), complex procedures (up to 17 pages), perception of unsustainable cost, anxiety on future sustainability, long time. Facilitators: relief after appeal won, Special Authority coverage (Canada), support from patient associations and FH registries.

Legend. LDL-C: Low-Density Lipoprotein Cholesterol; FH = Familial Hypercholesterolemia.

## Data Availability

The data supporting this research are available upon request from the corresponding authors for data protection reasons.

## Supplementary Materials

The following supporting information can be downloaded at: https://www.mdpi.com/article/10.3390/medicina62050843/s1, File S1: Preferred Reporting Items for Systematic reviews and Meta-Analyses extension for Scoping Reviews (PRISMA-ScR) Checklist, File S2. Search strategy.

## References

[1] World Health Organization, WHO Cardiovascular Diseases (CVDs). https://www.who.int/news-room/fact-sheets/detail/cardiovascular-diseases-(cvds).

[2] Wang X., Liang G. (2026). The global burden of ischemic heart disease attributable to trans fatty acid in 204 countries and territories, 1990–2023: A systematic analysis for the Global Burden of Disease Study 2023. Hum. Genom..

[3] Münzel T., Lüscher T., Kramer C.M., Churchwell K., Mbakwem A., Rajagopalan S. (2026). Environmental Stressors and Cardiovascular Health: Acting Locally for Global Impact in a Changing World: A Statement of the European Society of Cardiology, the American College of Cardiology, the American Heart Association, and the World Heart Federation. Circulation.

[4] Mach F., Koskinas K.C., Roeters van Lennep J.E., Tokgözoğlu L., Badimon L., Baigent C., Benn M., Binder C.J., Catapano A.L., De Backer G.G. (2025). 2025 Focused Update of the 2019 ESC/EAS Guidelines for the management of dyslipidaemias. Eur. Heart J..

[5] Blais J.E., Wei Y., Yap K.K.W., Alwafi H., Ma T.T., Brauer R., Lau W.C.Y., Man K.K.C., Siu C.W., Tan K.C.B. (2021). Trends in lipid-modifying agent use in 83 countries. Atherosclerosis.

[6] Marston N.A., Giugliano R.P., Im K., Silverman M.G., O’Donoghue M.L., Wiviott S.D., Ference B.A., Sabatine M.S. (2019). Association Between Triglyceride Lowering and Reduction of Cardiovascular Risk Across Multiple Lipid-Lowering Therapeutic Classes: A Systematic Review and Meta-Regression Analysis of Randomized Controlled Trials. Circulation.

[7] Orkaby A.R., Driver J.A., Ho Y.L., Lu B., Costa L., Honerlaw J., Galloway A., Vassy J.L., Forman D.E., Gaziano J.M. (2020). Association of Statin Use With All-Cause and Cardiovascular Mortality in US Veterans 75 Years and Older. JAMA.

[8] Yang C., Wu Y.J., Qian J., Li J.J. (2023). Landscape of Statin as a Cornerstone in Atherosclerotic Cardiovascular Disease. Rev. Cardiovasc. Med..

[9] Mach F., Visseren F.L.J., Cater N.B., Salhi N., Soronen J., Ray K.K., Delgado V., Jukema J.W., Laufs U., Zamorano J.L. (2024). Addressing residual risk beyond statin therapy: New targets in the management of dyslipidaemias—A report from the European Society of Cardiology Cardiovascular Round Table. J. Clin. Lipidol..

[10] McPherson R., Adreak N., Sharma A. (2024). Medications for Lipid Control: Statins vs Newer Drugs. Can. J. Cardiol..

[11] Agnello F., Ingala S., Laterra G., Scalia L., Barbanti M. (2024). Novel and Emerging LDL-C Lowering Strategies: A New Era of Dyslipidemia Management. J. Clin. Med..

[12] Hao Q., Aertgeerts B., Guyatt G., Bekkering G.E., Vandvik P.O., Khan S.U., Rodondi N., Jackson R., Reny J.L., Al Ansary L. (2022). PCSK9 inhibitors and ezetimibe for the reduction of cardiovascular events: A clinical practice guideline with risk-stratified recommendations. BMJ.

[13] Kim K., Ginsberg H.N., Choi S.H. (2022). New, Novel Lipid-Lowering Agents for Reducing Cardiovascular Risk: Beyond Statins. Diabetes Metab. J..

[14] Petrelli F., Cangelosi G., Nittari G., Pantanetti P., Debernardi G., Scuri S., Sagaro G.G., Nguyen C.T.T., Grappasonni I. (2021). Chronic Care Model in Italy: A narrative review of the literature. Prim. Health Care Res. Dev..

[15] Fischer L., Smeets R.G.M., Rijken M., Elissen A.M.J. (2025). Barriers and facilitators to integrated primary care from the perspective of people with chronic conditions and multiple care needs: A scoping review. Health Policy.

[16] Zezai D., van Rensburg A.J., Babatunde G.B., Kathree T., Cornick R., Levitt N., Fairall L.R., Petersen I. (2024). Barriers and facilitators for strengthening primary health systems for person-centred multimorbid care in low-income and middle-income countries: A scoping review. BMJ Open.

[17] Khatri R.B., Wolka E., Nigatu F., Zewdie A., Erku D., Endalamaw A., Assefa Y. (2023). People-centred primary health care: A scoping review. BMC Prim. Care.

[18] Chegini Z., Arab-Zozani M., Shariful Islam S.M., Tobiano G., Abbasgholizadeh Rahimi S. (2021). Barriers and facilitators to patient engagement in patient safety from patients and healthcare professionals’ perspectives: A systematic review and meta-synthesis. Nurs. Forum.

[19] Won M.H., Hwang I.S., Shin S.H. (2024). Influence of patient safety perception and attitude on inpatients’ willingness to participate in patient safety: An observation study. Medicine.

[20] Ray K.K., Landmesser U., Leiter L.A., Kallend D., Dufour R., Karakas M., Hall T., Troquay R.P., Turner T., Visseren F.L. (2017). Inclisiran in Patients at High Cardiovascular Risk with Elevated LDL Cholesterol. N. Engl. J. Med..

[21] Sabatine M.S. (2022). PCSK9 inhibitors: What we know, what we should have understood, and what is to come. Eur. Heart J..

[22] Ballantyne C.M., Graham T.E., Van Anglen L.J., Iteld B.J., Serota H., Niu X., McElligott S., Hanna K.E., Varisco T.J. (2025). Real-World Adherence and Effectiveness of Inclisiran in Lowering LDL-C: Results from 1 Year of Follow-Up. Cardiol. Ther..

[23] Taub P.R., Gutierrez A., Wewers D., Garcia Cantu E., Cao H., Deck C., Lesogor A., Ott D., Mena-Madrazo J., Zang X. (2025). Safety and Lipid-Lowering Efficacy of Inclisiran Monotherapy in Patients Without ASCVD: The VICTORION-Mono Randomized Clinical Trial. J. Am. Coll. Cardiol..

[24] Junqueira D., Molenkamp V., Goel R., Ikuemonisan J., Campbell P.J. (2026). Implementation strategies to optimize the use of nonstatin add-on lipid-lowering therapies in individuals with dyslipidemia: A systematic review. J. Clin. Lipidol..

[25] Azari S., Pourasghari H., Rezaei M.A., Behzadifar M., Salehbeigi S., Rajaei S., Jafarzadeh D., Soleimanpour S., Tajdini M. (2025). Fair pricing, fair access; a systematic review of cost-effectiveness of new hyperlipidemia injectable medication in developing countries. Cost. Eff. Resour. Alloc..

[26] Bäck M., Antoniou S., Butler T., Dendale P., Greco A., Hansen D., Hill L., Neubeck L., Rand A., Theuns D.A.M.J. (2026). A multidisciplinary approach to reduce the burden of cardiovascular disease, with special reference to the allied professionals’ perspective: A clinical consensus statement by the European Society of Cardiology Task Force on Allied Professionals with contributions from the Association of Cardiovascular Nursing and Allied Professions, the Association for Acute CardioVascular Care, the European Association of Percutaneous Cardiovascular Interventions, the European Association of Preventive Cardiology, the European Heart Rhythm Association, and the Heart Failure Association of the European Society of Cardiology. Eur. J. Cardiovasc. Nurs..

[27] Aromataris E., Lockwood C., Porritt K., Pilla B., Jordan Z. (2024). JBI Manual for Evidence Synthesis.

[28] Tricco A.C., Lillie E., Zarin W., O’Brien K.K., Colquhoun H., Levac D., Moher D., Peters M.D., Horsley T., Weeks L. (2018). PRISMA extension for scoping reviews (PRISMA-ScR): Checklist and explanation. Ann. Intern. Med..

[29] Ouzzani M., Hammady H., Fedorowicz Z., Elmagarmid A. (2016). Rayyan—A web and mobile app for systematic reviews. Syst. Rev..

[30] Braun V., Clarke V. (2006). Using Thematic Analysis in Psychology. Qual. Res. Psychol..

[31] Kosmas C.E., Silverio D., Ovalle J., Montan P.D., Guzman E. (2018). Patient adherence, compliance, and perspectives on evolocumab for the management of resistant hypercholesterolemia. Patient Prefer. Adherence.

[32] Baum S.J., Toth P.P., Underberg J.A., Jellinger P., Ross J., Wilemon K. (2017). PCSK9 inhibitor access barriers—Issues and recommendations: Improving the access process for patients, clinicians and payers. Clin. Cardiol..

[33] Wong N.D., Bang M., Block R.C., Peterson A.L.H., Karalis D.G. (2021). Perceptions and Barriers on the Use of Proprotein Subtilisin/Kexin Type 9 Inhibitors in Heterozygous Familial Hypercholesterolemia (From a Survey of Primary Care Physicians and Cardiologists). Am. J. Cardiol..

[34] Lee G.A., Durante A., Baker E.E., Vellone E., Caggianelli G., Dellafiore F., Khan M., Khatib R. (2024). Healthcare professionals’ perspectives on the use of PCSK9 inhibitors in cardiovascular disease: An in-depth qualitative study. Eur. J. Cardiovasc. Nurs..

[35] Baig S., Mughal S., Murad Y., Virdee M., Jalal Z. (2024). Exploring the Perceptions and Behaviours of UK Prescribers Concerning Novel Lipid-Lowering Agent Prescriptions: A Qualitative Study. Pharmacy.

[36] Siemens R., Pryjma M., Buchkowsky S., Barry A.R. (2024). Real-world effectiveness of monoclonal antibody inhibitors of PCSK9 in patients with heterozygous familial hypercholesterolemia: A retrospective cohort study. Pharmacotherapy.

[37] Mulder J.W.C.M., Galema-Boers A.M.H., Kranenburg L.W., Redekop K., Roeters van Lennep J.E. (2025). PCSK9 inhibitor experiences and preferences of patients and healthcare professionals in decision-making: A mixed methods study. Atherosclerosis.

[38] Religioni U., Barrios-Rodríguez R., Requena P., Borowska M., Ostrowski J. (2025). Enhancing Therapy Adherence: Impact on Clinical Outcomes, Healthcare Costs, and Patient Quality of Life. Medicina.

[39] George M., Maurina I., Schutte A.E. (2026). Therapeutic Adherence in Cardiovascular Diseases: Insights from the Patient and Physician-A Narrative Review. Adv. Ther..

[40] Lambrinou E., Kyriakou M., Lakatamitou I., Angus N., Khatib R., Vellone E., Barrowcliff A., Hansen T.B., Lee G.A. (2020). An integrative review on facilitators and barriers in delivering and managing injectable therapies in chronic conditions: A part of the ACNAP project ‘injectable medicines among patients with cardiovascular conditions’. Eur. J. Cardiovasc. Nurs..

[41] Lutfian L., Wardika I.J., Mukminin M.A., Zamroni A.H., Rizanti A.P., Chandra I.N., Widyaningtyas R., Maressa A., Maulana S. (2025). Effectiveness of health education in improving treatment adherence among patients with chronic communicable diseases: A systematic review and meta-analysis. Trop. Med. Int. Health.

[42] Iversen H.W., Riley H., Råbu M., Lorem G.F. (2025). Building and sustaining therapeutic relationships across treatment settings: A qualitative study of how patients navigate the group dynamics of mental healthcare. BMC Psychiatry.

[43] McGill E., Er V., Penney T., Egan M., White M., Meier P., Whitehead M., Lock K., Anderson de Cuevas R., Smith R. (2021). Evaluation of public health interventions from a complex systems perspective: A research methods review. Soc. Sci. Med..

[44] D’Alleva A., Leigheb F., Rinaldi C., Di Stanislao F., Vanhaecht K., De Ridder D., Bruyneel L., Cangelosi G., Panella M. (2019). Achieving quadruple aim goals through clinical networks: A systematic review. J. Healthc. Qual. Res..

[45] Alqahtani N.S. (2025). Lifestyle Counseling in Primary Care: Effectiveness, Strategies, and Clinical Implications. Int. J. Gen. Med..

[46] McAleavey A.A., de Jong K., Nissen-Lie H.A., Boswell J.F., Moltu C., Lutz W. (2024). Routine Outcome Monitoring and Clinical Feedback in Psychotherapy: Recent Advances and Future Directions. Adm. Policy Ment. Health.

[47] McCarron T.L., Noseworthy T., Moffat K., Wilkinson G., Zelinsky S., White D., Hassay D., Lorenzetti D.L., Marlett N.J. (2019). Understanding the motivations of patients: A co-designed project to understand the factors behind patient engagement. Health Expect..

[48] Bandhu D., Mohan M.M., Nittala N.A.P., Jadhav P., Bhadauria A., Saxena K.K. (2024). Theories of motivation: A comprehensive analysis of human behavior drivers. Acta Psychol..

[49] Choudry M., Ganti L. (2024). Exploration of the Motivational Factors that Influence the Maintenance of Health. Health Psychol. Res..

[50] Murphy J., Beauchamp N., Sun K.J., Lau B.D., Wilson R.F., Lobner K., Conway S.J., Hill P.M., Johnson P.T. (2026). Adverse effects of health plan prior authorization on clinical effectiveness and patient outcomes: A systematic review. Am. J. Med..

[51] Patel S., Huang M., Miliara S. (2025). Understanding Treatment Adherence in Chronic Diseases: Challenges, Consequences, and Strategies for Improvement. J. Clin. Med..

[52] Munuera Gómez P., Armadans Tremolosa I. (2024). Health mediation as an alternative means of conflict resolution in the practice of medicine in turbulent times: An update. Med. Clin..

[53] Grover S., Fitzpatrick A., Azim F.T., Ariza-Vega P., Bellwood P., Burns J., Burton E., Fleig L., Clemson L., Hoppmann C.A. (2022). Defining and implementing patient-centered care: An umbrella review. Patient Educ. Couns..

[54] Edgman-Levitan S., Schoenbaum S.C. (2021). Patient-centered care: Achieving higher quality by designing care through the patient’s eyes. Isr. J. Health Policy Res..

[55] Pantanetti P., Cangelosi G., Alberti S., Di Marco S., Michetti G., Cerasoli G., Di Giacinti M., Coacci S., Francucci N., Petrelli F. (2024). Changes in body weight and composition, metabolic parameters, and quality of life in patients with type 2 diabetes treated with subcutaneous semaglutide in real-world clinical practice. Front. Endocrinol..

[56] Moiz A., Filion K.B., Tsoukas M.A., Yu O.H.Y., Peters T.M., Eisenberg M.J. (2025). The expanding role of GLP-1 receptor agonists: A narrative review of current evidence and future directions. EClinicalMedicine.

[57] Thomsen R.W., Mailhac A., Løhde J.B., Pottegård A. (2025). Real-world evidence on the utilization, clinical and comparative effectiveness, and adverse effects of newer GLP-1RA-based weight-loss therapies. Diabetes Obes. Metab..

[58] Cangelosi G., Mancin S., Pantanetti P., Nguyen C.T.T., Morales Palomares S., Biondini F., Sguanci M., Petrelli F. (2024). Lifestyle Medicine Case Manager Nurses for Type Two Diabetes Patients: An Overview of a Job Description Framework—A Narrative Review. Diabetology.

[59] Cangelosi G., Grappasonni I., Pantanetti P., Scuri S., Garda G., Cuc Thi Thu N., Petrelli F. (2022). Nurse Case Manager Lifestyle Medicine (NCMLM) in the Type Two Diabetes patient concerning post COVID-19 Pandemic management: Integrated-Scoping literature review. Ann. Ig..

[60] Saultry B., Watts J.J., Mohebbi M., Bohingamu Mudiyanselage S., Lotfaliany M., Hutchinson A. (2022). Prioritising Responses Of Nurses To deteriorating patient Observations (PRONTO): A pragmatic cluster randomised controlled trial evaluating the effectiveness of a facilitation intervention on recognition and response to clinical deterioration. BMJ Qual. Saf..

[61] Mun M., Park Y., Hwang J., Woo K. (2024). Types and Effects of Telenursing in Home Health Care: A Systematic Review and Meta-Analysis. Telemed. J. E Health.

[62] Huang Z., Liu T., Chair S.Y. (2022). Effectiveness of nurse-led self-care interventions on self-care behaviors, self-efficacy, depression and illness perceptions in people with heart failure: A systematic review and meta-analysis. Int. J. Nurs. Stud..

